# Toward Intraoperative Visual Intelligence: Real-Time Surgical Instrument Segmentation for Enhanced Surgical Monitoring

**DOI:** 10.3390/healthcare12111112

**Published:** 2024-05-29

**Authors:** Mostafa Daneshgar Rahbar, George Pappas, Nabih Jaber

**Affiliations:** Department of Electrical and Computer Engineering, Lawrence Technological University, Southfield, MI 48075, USA; gpappas@ltu.edu (G.P.); njaber@ltu.edu (N.J.)

**Keywords:** intraoperative surgery, monitoring surgical scene, convolutional neural network, U-Net, data augmentation, surgical tools segmentation, computer vision, image processing

## Abstract

Background: Open surgery relies heavily on the surgeon’s visual acuity and spatial awareness to track instruments within a dynamic and often cluttered surgical field. Methods: This system utilizes a head-mounted depth camera to monitor surgical scenes, providing both image data and depth information. The video captured from this camera is scaled down, compressed using MPEG, and transmitted to a high-performance workstation via the RTSP (Real-Time Streaming Protocol), a reliable protocol designed for real-time media transmission. To segment surgical instruments, we utilize the enhanced U-Net with GridMask (EUGNet) for its proven effectiveness in surgical tool segmentation. Results: For rigorous validation, the system’s performance reliability and accuracy are evaluated using prerecorded RGB-D surgical videos. This work demonstrates the potential of this system to improve situational awareness, surgical efficiency, and generate data-driven insights within the operating room. In a simulated surgical environment, the system achieves a high accuracy of 85.5% in identifying and segmenting surgical instruments. Furthermore, the wireless video transmission proves reliable with a latency of 200 ms, suitable for real-time processing. Conclusions: These findings represent a promising step towards the development of assistive technologies with the potential to significantly enhance surgical practice.

## 1. Introduction

Traditionally, surgery relies solely on the surgeon’s direct view for guidance. However, the past decade has witnessed a surge in the automated analysis of surgical video data. Surgical video analysis techniques offer surgeons various benefits, such as generating post-operative reports [1,2], evaluating surgical skills for training purposes [3], and creating educational content. Furthermore, real-time video analysis holds promise for intraoperative communication with surgeons, including the development of automated warning or recommendation systems based on the real-time recognition of surgical tasks, steps, or gestures [4,5,6]. These systems could potentially improve safety by detecting deviations from standard surgical procedures. However, a major challenge remains in interpreting surgical video data effectively: the accurate detection of all surgical instruments. These instruments come in a wide variety of shapes and sizes and often appear partially obscured within the surgical field. Many studies have focused on tackling this problem of surgical instrument detection. In the realm of minimally invasive surgery, particularly laparoscopy, technological advancements have revolutionized procedures. Laparoscopic surgeons rely on endoscopic cameras for viewing the surgical field, allowing for minimally invasive interventions. Similar technological progress can potentially improve the visualization and coordination of intricate surgical maneuvers in traditional open surgeries, enhancing surgical precision and efficiency on standard video monitors. Open surgery relies heavily on the surgeon’s visual acuity and spatial awareness to track instruments within a dynamic and often cluttered surgical field. Maintaining focus and instrument identification can be challenging, especially during long procedures or intricate maneuvers.

This project addresses this challenge by developing a novel visual intelligence system utilizing an optical approach for real-time surgical instrument tracking. The significance of this project lies in its ability to monitor the open surgery scene using a head-mounted depth camera. The system then segments surgical instruments in real-time from the captured video stream. This real-time segmentation offers several key advantages: **(1) Enhanced Situational Awareness:** By segmenting instruments, the system can visually highlight them, aiding the surgeon in quickly identifying and tracking their location within the surgical field. This is particularly beneficial during minimally invasive procedures or situations with obscured views. **(2) Improved Surgical Efficiency:** real-time instrument tracking can potentially streamline surgical workflow by reducing the time spent searching for or confirming instrument location. **(3) Potential for Data-Driven Insights:** segmented instrument data can be further analyzed to provide valuable insights into surgical techniques, instrument usage patterns, and potentially even identify potential errors or complications.

This project focuses on achieving real-time instrument segmentation through a system design that incorporates the following: **(a) Head-Mounted Depth Camera:** this allows for a hands-free approach to capturing a surgical scene from the surgeon’s perspective. **(b) Wireless Video Transmission:** the real-time transmission of the captured video stream to a high-performance computer workstation enables the complex image processing required for segmentation. **(c) Real-Time Instrument Segmentation:** the system segments surgical instruments from the video stream on the high-performance computer, providing immediate feedback to the surgeon. By achieving these goals, this project contributes to the advancement of intelligent surgical assistance systems, aiming to improve the overall safety, efficiency, and potentially the quality of open surgical procedures. The data collected by this system extend beyond immediate intraoperative safety. They can be used to establish safety volumes and define safe working zones for instruments, aiding in risk assessments [7]. They also can be utilized to develop smart glass augmentations and provide visual cues and warnings to improve surgical precision [8]. Last but not least, they can be used to enhance surgical training by offering feedback on techniques and movement patterns for inexperienced surgeons, facilitating faster learning [9,10].

The importance of monitoring open surgery scenes and segmenting surgical instruments is underscored by the need to enhance visualization and control during procedures. Hasan [11] and Hajj [12] both highlight the significance of segmenting and removing surgical instruments to improve the surgeon’s view and facilitate automated instrument detection. Payandeh [13] and Panait [14] further emphasize the potential for image processing and video image enhancement to enhance surgical skills and facilitate finer maneuvers. However, the use of video images on standard monitors in open surgeries may lead to longer task performance [15]. Reiner [16] and Padoy [17] explore the potential of reproducing stereoscopic images and monitoring surgical procedures, respectively, to further improve visualization and workflow.

A range of studies have explored the use of advanced technologies in surgical settings. Islam [18] and Shvets [19] both developed deep learning-based systems for real-time instrument segmentation, with Islam’s system outperforming existing algorithms. Fan [20] and Novotny [21] focused on 3D visualization and tracking, with Fan’s system achieving a spatial position error of 0.38 ± 0.92 mm. Gering [22] and Dergachyova [23] integrated image fusion and interventional imaging and proposed a data-driven method for surgical phase segmentation and recognition, respectively. Su [24] and Zhao [25] both developed real-time segmentation and tracking systems, with Su’s algorithm achieving robust performance without GPU acceleration. These studies collectively demonstrate the potential of advanced technologies in enhancing surgical procedures.

Monitoring a surgical scene during open surgery using an optical approach is the main aim of this research. This work aims to develop and validate a real-time visual intelligence system for tracking and segmenting surgical instruments during open surgery. Instrument tracking accurately: tracking surgical instruments in the dynamic of an operating room is one of most important underlying challenges of the research. Instrument identification/segmentation: distinguishing surgical instruments from other objects in the surgical field (hands, tissues, etc.) is another problem that this work tackles. Real-time monitoring: providing surgeons with updated information quickly and minimizing delays that can hinder the surgical process is another important challenge that this research tries to find a solution for.

For rigorous validation, the system’s performance, reliability, and accuracy are evaluated using prerecorded RGB-D surgical videos. This work demonstrates the potential of this system to improve situational awareness, surgical efficiency, and generate data-driven insights within the operating room. In a simulated surgical environment, the system achieved a high accuracy of 85.5% in identifying and segmenting surgical instruments. Furthermore, the wireless video transmission proved reliable with a latency of 200 ms, suitable for real-time processing. These findings represent a promising step towards the development of assistive technologies, with the potential to significantly enhance surgical practice.

## 2. Materials and Methods

This system utilizes a head-mounted depth camera to monitor surgical scenes, providing both image data and depth information. The surgeon’s perspective facilitates a clear view of the surgical field. The video captured from this camera is scaled down, compressed using MPEG, and transmitted to a high-performance workstation via the RTSP (Real-Time Streaming Protocol), a reliable protocol designed for real-time media transmission. The received video undergoes preprocessing, including optical flow filtering and other image processing techniques, to enhance the relevant features. To segment surgical instruments, we employ a convolutional neural network (CNN) approach. Specifically, we utilize an enhanced U-Net with GridMask (EUGNet) [26] for its proven effectiveness in surgical tool segmentation.

### 2.1. Head-Mounted Depth Camera

The use of RGB-D depth cameras, such as the Intel RealSense series, offers significant advantages for real-time intraoperative surgery monitoring. The RGB component provides traditional visual information for instrument identification, while the depth component enables a more comprehensive understanding of the surgical field’s 3D structure. This depth information aids in accurately tracking instrument locations, even in complex or cluttered environments. Additionally, depth data can enhance instrument segmentation algorithms, improving their ability to distinguish surgical tools from the background and other objects with similar visual appearances. The integration of an RGB-D RealSense camera within a surgical monitoring system holds the potential to enhance procedural safety, efficiency, and the collection of valuable intraoperative data.

RGB-D RealSense technology can be useful for intraoperative surgery monitoring by providing the real-time 3D visualization and tracking of surgical instruments and anatomical structures [27]. It utilizes depth sensors like the Intel RealSense to capture color and depth data, enabling augmented reality overlays and enhanced navigation during procedures [28]. This real-time imaging modality complements traditional intraoperative imaging techniques like ultrasound and MRI, offering additional spatial awareness and guidance [29]. RGB-D sensing can track respiratory motion and the deformation of soft tissues, allowing for more precise targeting and compensation during interventions [27]. The combination of color and depth data provides an enhanced visualization of the surgical field, aiding in instrument navigation and the identification of critical structures [28]. Overall, RGB-D RealSense technology shows promise as an intraoperative imaging modality, offering real-time 3D guidance and augmented reality capabilities to improve surgical precision and safety.

Head-mounted RGB-D sensors like RealSense can be useful for intraoperative monitoring during posterior skull base surgery [30]. They allow the real-time visualization of surgical instruments and anatomical structures, providing enhanced guidance to the surgeon [31]. The system uniquely employs a head-mounted RGB-D camera, positioning it directly at the surgeon’s perspective. This setup provides a wider field of view and generates two crucial data streams: an RGB frame for visual context and a point cloud for 3D spatial analysis.

### 2.2. UP Squared Board

For data transfer, the system employs a UP Squared board. This compact computer acts as a wireless bridge, receiving the captured RGB video and point cloud data stream from the surgeon-mounted RealSense camera. The UP Squared board then transmits these data wirelessly to a high-performance workstation for real-time analysis. The use of the UP Squared board for wireless data transfer aligns with previous research on real-time remote monitoring systems. Velásquez-Aguilar and Yan both highlight the use of wireless communication for data acquisition and transmission, with Yan specifically focusing on a low-cost wireless bridge [32,33]. The real-time aspect of the system is further supported by [34], who presents a wireless solution for data acquisition in a real-time environment. The system’s ability to capture and calibrate video data is also in line with [35] on a low-cost hardware platform for video capture. The use of a single-chip microcomputer for network data transfer, as discussed by [35], could potentially enhance the efficiency of the system. Lastly, the system’s potential for high-efficiency video transmission is supported by [36] on a compressed sensing-based video transmission system.

### 2.3. High Performance Work Station

**Figure 1:**
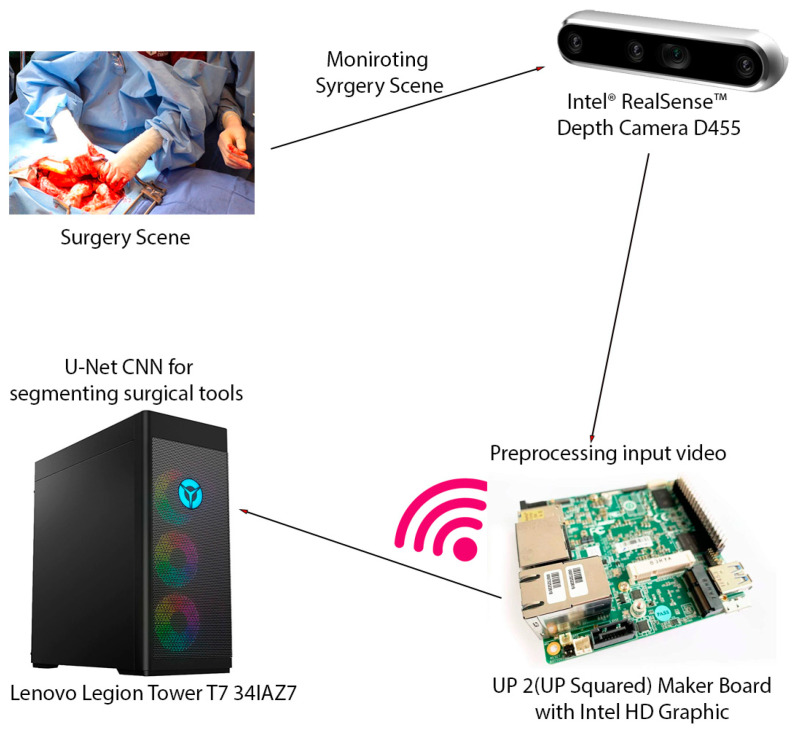
The intraoperative visual intelligence system is designed to provide real-time surgical instrument segmentation and tracking during open surgical procedures. This system leverages a multi-component hardware setup to capture, process, and analyze surgical scenes. The key hardware components of the system include Intel^®^ RealSense™ Depth Camera D455, UP 2 (UP Squared) Maker Board with Intel HD Graphic, Lenovo Legion Tower T7 341AZ7, and wireless transmission. This integrated hardware system enables the efficient capture, transmission, and processing of surgical video data, facilitating real-time instrument segmentation and tracking and ultimately enhancing surgical visualization and safety.

The video captured from the RealSense camera is preprocessed and transmitted to a high-performance workstation. This is a computer with a 13th Generation Intel Core™ i9-13900KF processor (E-cores up to 4.30 GHz P-cores up to 5.40 GHz) CPU and an NVIDIA GeForce RTX™ 4080 16GB GDDR6X GPU. The average inference time, encompassing data transfers between the CPU and GPU, was calculated over 1000 inferences. The video was then scaled down, compressed using MPEG, and transmitted to a high-performance workstation via the RTSP protocol, which is designed for reliable real-time media transmission. Upon reception, the video underwent preprocessing, including optical flow filtering and other image processing techniques, to enhance pertinent features. As it was mentioned earlier, to segment surgical instruments, we employ a convolutional neural network (CNN) approach. Specifically, we utilize an enhanced U-Net with GridMask (EUGNet) [26] for its proven effectiveness in surgical tool segmentation. Figure 1 summarizes the different components of the designed intraoperative visual intelligence system.

### 2.4. Middleware Framework

ROS is a middleware framework designed for robot software development. It facilitates communication between different robot components and offers tools for common robotic tasks like sensor data processing and motion control. Our system leverages specific ROS components to enhance certain aspects of a surgical instrument tracking project. ROS is used for data acquisition and visualization. Since Real Sense is an ROS-compatible camera, we leverage existing ROS drivers to streamline data acquisition and camera integration. ROS provides standardized message formats that simplify integrating video and depth data. ROS is also utilized for visualization. RViz, a powerful ROS visualization tool, could be used to display camera data alongside overlays indicating segmented instruments. This could provide real-time feedback during development or for intraoperative monitoring. Modular system design is another reason that ROS is selected as the middleware framework. Its node-based architecture is employed to structure parts of a system as ROS nodes. This promotes modularity, where image processing, instrument segmentation, and data transmission components can communicate via ROS topics and services. We find existing ROS packages for image processing or computer vision tasks that could integrate seamlessly into a system. Gazebo, a simulator often used with ROS, is used to create simulated surgical environments. This helped to evaluate algorithms or generate synthetic training data. Because of its extensive libraries and tools, ROS is excellent for the rapid prototyping of algorithms for tracking or segmentation.

To create an ROS (Robot Operating System) publish–subscribe diagram for a described system involving the segmentation of surgical tools and movement analysis using a head-mounted depth camera, we outline the different nodes and topics involved. Here is a structured plan for the nodes and the information flow in the system:

**1.**  **Camera Node**
  - **Purpose:** Captures video and depth data from a head-mounted depth camera.
  - **Publishes:**
    ○ **Topic:** /camera/image_raw (image data)
    ○ **Topic:** /camera/depth_raw (depth data)
**2.**  **Video Processing Node**
  - **Purpose:** Handles the reception, scaling down, and compression of video data.
  - **Subscribes:**
    ○ **Topic:** /camera/image_raw
    ○ **Processes:** Scales down and compresses video using MPEG and transmits it via the RTSP.
  - **Publishes:**
    ○ **Topic:** /video/compressed
**3.**  **Preprocessing Node**
  - **Purpose:** Processes the compressed video to prepare for segmentation.
  - **Subscribes:**
    ○ Topic: /video/compressed
  - **Processes:** Applies optical flow filtering and other image processing techniques.
  - **Publishes:**
    ○ Topic: /video/processed
**4.**  **Segmentation Node**
  - **Purpose:** Segments surgical instruments from the video.
  - **Subscribes:**
    ○ Topic: /video/processed
  - **Processes:** Uses a convolutional neural network (EUGNet with GridMask) for segmentation.
  - **Publishes:**
    ○ Topic: /video/segmented_tools
**5.**  **Movement Analysis Node**
  - **Purpose:** Analyzes the movement of segmented tools.
  - **Subscribes:**
    ○ **Topic:** /video/segmented_tools
  - **Processes:** Conducts movement analysis.
  - **Publishes:**
    ○ **Topic:** /tools/movement_analysis
**6.**  **Visualization Node**
  - **Purpose:** Visualizes the segmented tools and their movement analysis for monitoring and further analysis.
  - **Subscribes:**
    ○ **Topic:** /video/segmented_tools
    ○ **Topic:** /tools/movement_analysis

### 2.5. Surgical Instrument Segmentation Network

**Figure 2:**
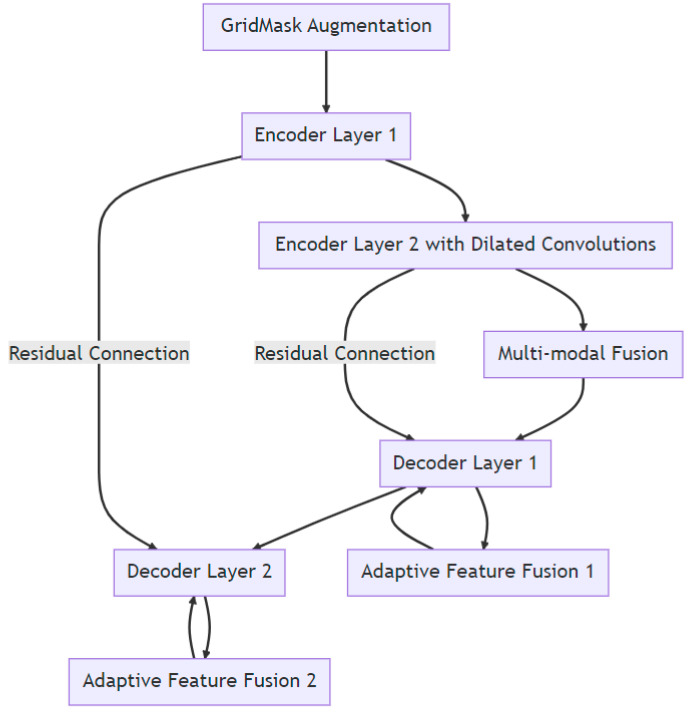
Enhanced U-Net with GridMask (EUGNet) architecture for robust real-time surgical instrument segmentation: leveraging deep contextual encoding, adaptive feature fusion, and GridMask data augmentation and data collection for algorithm evaluation.

An enhanced U-Net with GridMask (EUGNet), which incorporates GridMask augmentation to address U-Net’s limitations and is proposed in a previous work [26], is utilized to conduct surgical instrument segmentation. EUGNet features a deep contextual encoder, residual connections, class-balancing loss, adaptive feature fusion, a GridMask augmentation module, efficient implementation, and multi-modal fusion. These innovations enhance segmentation accuracy and robustness, making it well suited for medical image analysis. The GridMask algorithm, designed for improved pixel elimination, demonstrates its effectiveness in enhancing model adaptability to occlusions and local features, which are crucial in dynamic surgical environments. A rigorous evaluation of the framework’s robustness is conducted using a comprehensive dataset of robotic surgical scenarios and instruments. Employing the U-Net architecture as a baseline, the integration of GridMask as a data augmentation technique significantly enhances both segmentation accuracy and inference speed. These results highlight GridMask’s potential as a valuable tool for real-time instrument–tissue segmentation in robotic surgery. Figure 2 depicts a visual representation of EUGNet.

To demonstrate the robustness and generalization ability of the proposed robotic instrument segmentation framework, we employed a dataset featuring diverse surgical scenarios and instruments. This dataset included the Da Vinci Robotic (DVR) dataset [37] with four 45 s ex vivo training videos and six test videos (ranging from 15 to 60 s) showcasing two articulated instruments. Ground truth masks were automatically generated with manual correction, and videos were recorded using the dVRK open-source platform [38]. Additionally, we utilized open-source videos from the U.S. National Library of Medicine [39] depicting procedures like midline lobectomy, right superior line lobectomy, thoracotomy, thoracoscopic lung surgery, and prostatectomy with occurrences of “splash-like” bleeding. Also, we used datasets from the EndoVis challenge [40] for comprehensive evaluations. Finally, the Endoscapes Dataset [41] provided 201 laparoscopic videos richly annotated for scene segmentation, object detection, and a critical view of safety assessments. All videos across these datasets had a resolution of 720 × 567 and ran at 25 frames per second.

### 2.6. Baseline Method and Evaluation Protocol

U-Net is a popular choice for medical image analysis, particularly for segmenting surgical instruments. As a baseline for comparison, we employed a state-of-the-art U-Net architecture known for its effectiveness in segmenting robotic surgical tools. This choice leverages U-Net’s well-established capabilities for this task. To improve the model’s ability to generalize unseen data during training, we utilized random image selection. We prioritized comparing our proposed architecture to this baseline over achieving the highest possible segmentation scores. Additionally, GridMask data augmentation was applied to further enhance the model’s robustness. To avoid potential biases from the source dataset being introduced during transfer learning, we opted to train the model from scratch. In our experiments, the cyclical learning rate (CLR) bounds for the U-Net network were set to (10^−4^; 10^−2^). The quantitative metrics of choice to evaluate the predicted segmentations were the mean intersection over union (mean IoU) and mean Dice similarity coefficient (mean DSC):

(1) $$\bar{IoU}\left(\hat{y},y\right)=\frac{1}{K}\overset{K}{\underset{k=1}{\sum }}\frac{TP_{k}}{TP_{k}+FP_{k}+NF_{k}}\;$$

(2) $$\bar{DSC}\left(\hat{y},y\right)=\frac{1}{K}\overset{K}{\underset{k=1}{\sum }}\frac{2TP_{k}}{2TP_{k}+FP_{k}+NF_{k}}\;\;$$

where *K* = 2 (*k* = 0 background and *k* = 1 foreground), and $TP_{k}$, $FP_{k}$, and $NF_{k}$ represent true positives, false positives, and false negatives for class k, respectively.

All networks were trained and tested on a computer equipped with a 13th Generation Intel Core™ i9-13900KF processor (E-cores up to 4.30 GHz and P-cores up to 5.40 GHz) and an NVIDIA GeForce RTX™ 4080 16GB GDDR6X GPU. The reported inference times included data transfers between the CPU and GPU and were averaged over 1000 inferences.

The Intel RealSense D455 is an advanced stereo depth camera that extends the capabilities of its predecessors by providing greater accuracy and longer range sensing. Equipped with two high-resolution depth sensors and an RGB sensor, the D455 offers a depth range of up to 20 m, making it suitable for a variety of applications, from robotics and augmented reality to more complex scenarios like gesture recognition and people tracking. Its improved accuracy and wider baseline of 95 mm between the depth sensors enhance depth perception and reduce blind spots, thus providing more precise 3D imaging.

One of the standout features of the RealSense D455 is its built-in IMU (inertial measurement unit), which provides additional data points about device orientation and movement, enhancing the depth data with spatial awareness. This feature is particularly valuable for mobile applications, where understanding the device’s position and orientation in space is crucial. The D455 is designed to be plug and play, supporting both Windows and Linux environments, and integrates seamlessly with the Intel RealSense SDK 2.0, which offers a rich set of libraries and APIs to expedite development and integration. Whether used for creating interactive experiences, developing navigation systems for drones and robots, or for enhanced computer vision in complex environments, the Intel RealSense D455 provides developers and engineers with a powerful tool to bring depth-sensing capabilities to their projects.

The Intel Up Squared (or UP²) board is a compact and powerful single-board computer designed for a variety of applications in embedded computing, the Internet of Things (IoT), and edge computing spaces. It represents an evolution in the Up board series, offering significantly enhanced processing powers, flexibility, and connectivity compared to its predecessors.

The UP Squared board features Intel Apollo Lake processors, including Intel Celeron Intel^®^ Pentium^®^ J6426. This processor provide a balance between performance and power efficiency, making the board suitable for demanding tasks that also require low power consumption. It has 16GB DDR4 RAM and 64GB of eMMC storage, providing ample space and speed for various applications. Additionally, there is support for an M.2 SSD, enhancing its capabilities for storage-intensive applications. The board includes multiple USB ports (USB 3.0 and USB 2.0), Gigabit Ethernet, HDMI, and DisplayPort, facilitating a wide range of connectivity options for peripherals and displays. It also supports wireless connections via an M.2 slot that can host WiFi and Bluetooth modules. Due to its powerful features and multiple connectivity options, UP Squared is suitable for a wide range of applications such as digital signage, kiosks, IoT gateways, smart home devices, edge computing devices, and even as a development platform for AI and machine learning projects.

The Intel^®^ Wi-Fi 6 AX210 module provides a future-proof option that supports the latest WiFi 6 technology. It supports 802.11ax technology, which can significantly improve throughput and capacity, particularly in dense environments. It also offers Bluetooth 5.1 for extended range and capabilities. It is good for high-performance applications requiring the highest data rates and improved efficiency, such as streaming high-definition videos, gaming, or handling multiple wireless devices simultaneously [42].

## 3. Results

**Figure 3:**
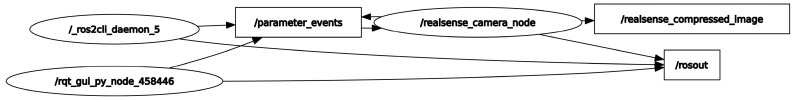
rqt_graph-generated ROS communication.

In this study, ROS 2 was deployed on a UP Squared board operating under a Linux Ubuntu environment to establish a sophisticated video processing framework. A custom video publisher node written in C++ was developed to interface with an Intel RealSense camera, which captures video data in real time. This node is responsible for the initial preprocessing and compression of the video data before publishing it to a designated ROS topic named realsense_compressed_image. A corresponding subscriber node, executed on a high-performance computing system as previously described, subscribes to the realsense_compressed_image topic. Both machines are configured within the same DDS (Data Distribution Service) domain, facilitating seamless intra-domain communication. This setup ensures that the video data are efficiently captured, processed, and transmitted between the nodes without significant latency or a loss of data integrity. Figure 3 in the manuscript illustrates the use of rqt_graph, a graphical tool provided by ROS for visualizing the computation graph of the system. rqt_graph effectively demonstrates the active nodes and their interconnections through topics within the ROS environment. This visualization is generated subsequent to the activation of the aforementioned nodes on both computers, providing a clear and intuitive representation of the dynamic interactions and data flow within the network. The integration of ROS 2 with the UP Squared board and RealSense camera showcases a robust platform for real-time video processing applications, highlighting the system’s scalability and flexibility in handling complex computational tasks across distributed nodes. The wireless video transmission proved reliable with a latency of 200 ms, suitable for real-time processing.

Our proposed data augmentation technique, GridMask, significantly improved the performance of the U-Net architecture on the EndoVis testing set. Metrics including balanced accuracy (foreground), the mean intersection over union (IoU), and the mean Dice similarity coefficient (DSC) all demonstrated substantial gains (specific values are shown in Table 1). Furthermore, GridMask drastically reduced the inference time from 0.163 ms for the baseline U-Net to 0.097 ms for the U-Net with GridMask. This reduction facilitates real-time instrument–tissue segmentation, achievable at approximately 29 frames per second. Qualitative analysis reveals that our method using GridMask achieves better adherence to the boundaries of left-handed surgical instruments.

**Figure 4:**
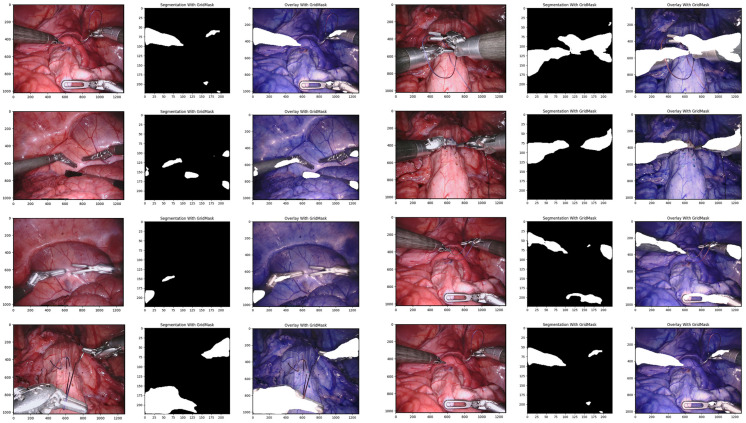
Qualitative comparison of our proposed convolutional architectures with GridMask data augmentation.

Using GridMask data augmentation alongside the U-Net architecture, we achieved substantial improvements on the Endoscape dataset [41]. Metrics such as balanced accuracy, IoU, and mean DSC showed significant gains (see Table 1 for specific values). Notably, GridMask drastically reduced the inference time to 0.097 ms, making the approach suitable for real-time instrument–tissue segmentation at approximately 29 fps. Qualitative analysis (see Figure 4) demonstrates the enhanced adherence to tool boundaries when using GridMask, especially for left-handed surgical instruments.

**Figure 5:**
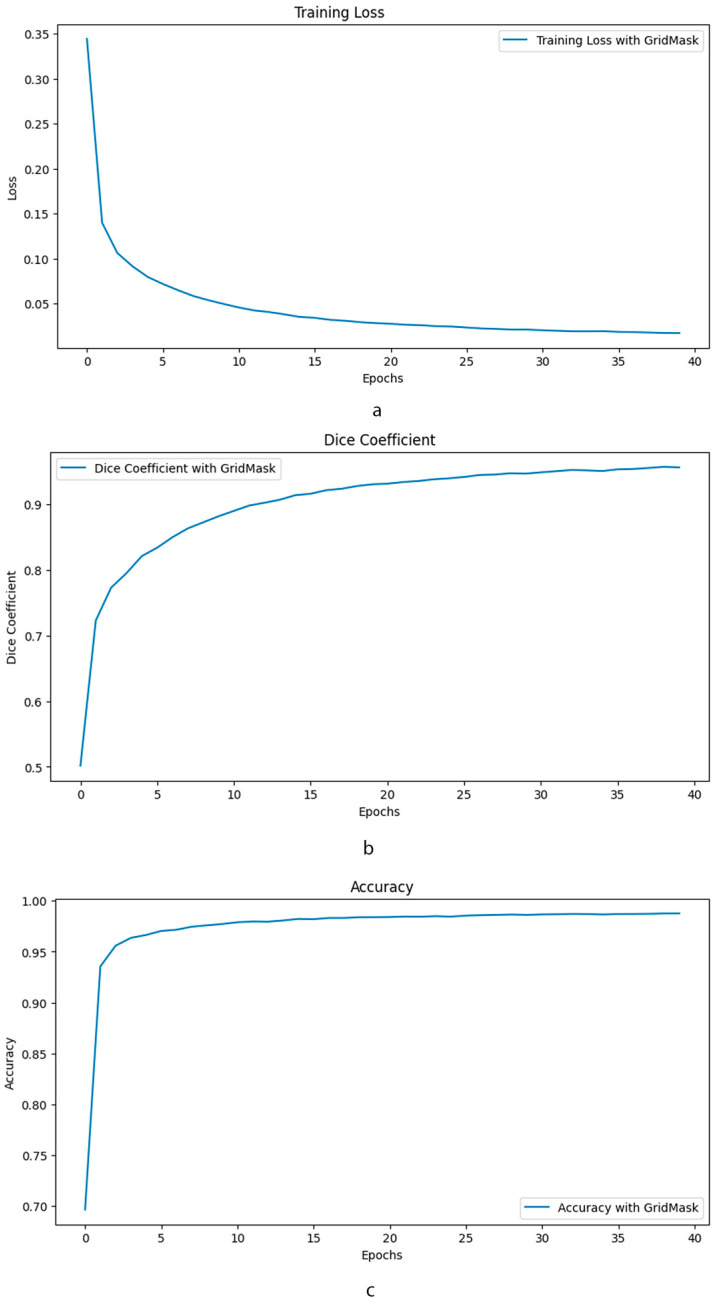
Comparative performance of enhanced U-Net with and without GridMask data augmentation. This composite image presents a comprehensive evaluation of the impact of GridMask data augmentation on the performance of an enhanced U-Net model. The evaluation is conducted over 40 epochs and focuses on three key performance indicators: (**a**) Training Loss: The graph illustrates the evolution of training loss over the course of training. (**b**) Dice Coefficient: This metric assesses the overlap between the predicted segmentation and the ground truth. (**c**) Accuracy: The overall accuracy of the model is presented.

The training loss curve in Figure 5 demonstrates a stable decline over 40 epochs when using GridMask, indicating good model generalization and minimal overfitting. This suggests the model effectively learns from the training data without memorizing specific details. Furthermore, accuracy metrics fall within the “excellent” range [43], implying the model’s predictions are highly reliable. The Dice coefficient, a crucial metric for segmentation tasks, also exhibits strong performance with GridMask, falling within the “excellent” range [44]. Notably, the consistently high Dice coefficient indicates a superior overlap between the model’s predicted segmentation masks and the ground truth labels. Collectively, these findings suggest that GridMask data augmentation significantly improves U-Net’s ability to learn robust and accurate segmentation capabilities.

## 4. Discussion

In summary, this project aimed to enhance surgical procedures by developing a system that uses a head-mounted camera to track and identify surgical tools in real-time during open surgeries. This is achieved by transmitting videos from the surgeon’s perspective to a powerful computer that analyzes the images and identifies the tools. The system has been rigorously tested and found to be highly accurate in identifying tools in simulated surgical environments. This technology not only has the potential to improve the safety and efficiency of surgeries, but it can also be used to gather valuable data for training purposes and to develop new tools that further enhance surgical precision.

A range of studies have explored the use of head-mounted cameras for real-time monitoring in the operating room. The authors of [45,46] highlight the potential of smartphone and GoPro cameras, respectively, for capturing high-quality intraoperative footage. In [47], an overview of the advantages and disadvantages of different video capture methods, including head-mounted cameras, is provided. Based on these surveys, this technology is still a work-in-progress, and when it comes to its applicability in the operating room, it requires further fine-tuning to optimize its utility. Our proposed method is successfully utilized to monitor and capture higher-quality videos to monitor surgical scenes. It also provides an additional piece of information about the depth of surgical scenes. As one possible extension of this work, the depth information can be converted to a point cloud object and be transmitted to a high-performance system to provide information for the refined segmentation of surgical instruments. The instance segmentation of point clouds is extremely important since the quality of segmentation affects the performance of subsequent algorithms. The point cloud captured by an RGB-D sensor can go through the instance segmentation process by applying the deep learning method YOLACT++ to instance segment the color image first and then matching the instance information with the point cloud [48].

This work presents a surgical instrument segmentation system that leverages a distributed ROS 2 architecture for real-time data transmission and processing. The system employs a head-mounted depth camera at the surgical site, capturing high-quality RGB videos along with corresponding depth information. These data are transmitted, potentially wirelessly, using a reliable protocol like the RTSP (Real-Time Streaming Protocol), to a high-performance workstation for real-time instrument segmentation analysis. ROS 2 facilitates communication between the camera and the workstation, enabling a modular and scalable architecture for efficient data handling.

In comparison to our previous results in [26], downscaling captured videos at the surgeon’s site presents a potential reduction in the accuracy of U-Net surgical instrument segmentation. While a reduced video resolution improves transmission efficiency and potentially processing speeds, which are essential for real-time applications, it also results in a loss of fine visual details. This loss of detail can hinder the model’s ability to precisely delineate tool boundaries, especially for small or thin instruments. Overlapping instruments may become harder to distinguish, and textural details useful for classification could be lost. While U-Net’s skip connections offer some resilience to downscaling, excessive resolution reductions can limit their effectiveness. Understanding and mitigating these impacts is important. Experimentation is necessary to find the optimal balance between acceptable downscaling levels and segmentation accuracy, considering the specific surgical tasks of a project.

The potential for the large-scale application of this technology is indeed a key consideration. While the current validation focused on pre-recorded videos in specific surgical scenarios, the underlying principles and architecture of the system are designed to be adaptable to various surgical environments. The deep learning model at the core of the instrument segmentation process can be trained on diverse datasets encompassing a wider range of surgical procedures, including neurosurgery and general, urologic, gynecologic, and orthopedic surgeries. This would allow the system to generalize and effectively segment instruments in various contexts. However, it is important to acknowledge that each surgical specialty presents unique challenges due to variations in instruments, anatomical structures, and surgical techniques. Therefore, while the fundamental framework of the system remains applicable, tailored training and fine-tuning may be necessary to ensure optimal performance in each specific surgical domain. As we discuss in the paper, the adaptability and generalizability of the system are areas of ongoing research. Our future work will focus on expanding the training datasets and refining the model to accommodate a wider range of surgical scenarios, ultimately making this technology a valuable asset across various surgical specialties.

In conclusion, this research presents a promising step toward the future of surgical assistance systems. The developed system, capable of real-time surgical instrument segmentation and tracking, holds the potential to enhance surgical accuracy, efficiency, and safety by providing surgeons with real-time visual feedback and data-driven insights.

For healthcare professionals, the benefits are manifold: **(a) Improved Surgical Precision:** by accurately identifying and tracking instruments within the surgical field, the system can help surgeons maintain better control and precision, potentially leading to better surgical outcomes. **(b) Enhanced Situational Awareness:** the system’s ability to provide real-time visual feedback can enhance the surgeon’s awareness of the surgical field, reducing the risk of accidental injury and improving the overall safety. **(c) Data-Driven Insights:** the data generated by the system can be invaluable for post-operative analysis, surgical training, and the development of future surgical technologies.

However, despite the promising results, it is important to acknowledge potential limitations: **(a) Cost and Accessibility:** the high-performance computing and advanced processing technologies required for this system may be expensive and not readily accessible to all healthcare facilities. **(b) Integration Challenges:** integrating this new technology into existing surgical workflows and infrastructure could pose challenges, especially in older or less technologically equipped facilities. **(c) Adaptability Concerns:** older surgeons who are accustomed to traditional surgical practices may find it difficult to adapt to using a head-mounted camera and relying on real-time visual feedback. **(d) Procedural Limitations:** the system may not be suitable for all types of surgical procedures, particularly those that require an extensive manipulation of tissues or organs outside the camera’s field of view. **(e) Attention Diversion:** the use of a head-mounted camera and real-time visual feedback could potentially divert the surgeon’s attention away from the surgical field, necessitating careful training and adaptation. In light of these considerations, further research and development are needed to address these limitations and ensure the seamless integration of this technology into the diverse landscape of surgical practice. While the future of surgical assistance systems is promising, a careful consideration of these factors will be crucial for the successful and widespread adoption of this innovative technology.

## 5. Conclusions

In this research, we demonstrated a novel intraoperative visual intelligence system that enhances surgical monitoring by providing the real-time segmentation of surgical instruments. This advancement leverages cutting-edge technologies, including head-mounted depth cameras and convolutional neural networks, to significantly improve the visibility and tracking of surgical tools, thereby enhancing surgical precision and safety. The system’s ability to provide real-time feedback to surgeons is a critical development, particularly in complex surgeries where visibility is compromised. Our results indicate high accuracy in instrument identification and segmentation, demonstrating the system’s potential to not only support surgeons in real time but also to serve as a valuable training tool for surgical education.

Looking forward, the integration of such technologies promises to revolutionize the operating room, reducing surgical errors and improving patient outcomes. This work lays the groundwork for further innovations in surgical procedures and offers a glimpse into the future of automated and enhanced surgical environments. Further studies and refinements will likely focus on optimizing the system’s accuracy and responsiveness, expanding its application to various surgical contexts, and integrating deeper learning and adaptive algorithms to handle an even broader array of surgical instruments and conditions.

## Figures and Tables

**Table 1:** Quantitative results for segmentation of non-rigid robotic instruments in testing set videos. IoU stands for intersection over union and DSC for Dice similarity coefficient. The means are performed over classes, and the results presented are averaged across testing frames.

Network	Inference Time (ms/fps)	Balanced Accuracy (fg.)	Mean IoU	Mean DSC
EUGNet with EndoVis Dataset	30.2/25.2	89.3%	84.6%	85.5%
EUGNet with Endoscape Dataset	31.7/26.7	84.3%	82.6%	81.5%

## Data Availability

The data presented in this study are available at https://universe.roboflow.com/models/instance-segmentation (accessed on 2 February 2024) and https://www.ncbi.nlm.nih.gov/pmc/articles/PMC6462551/figure/vid/ (accessed on 5 March 2024).
